# Endonasal endoscopic treatment of a rare advanced Teratocarcinosarcoma of skull base

**DOI:** 10.1016/j.bjorl.2025.101707

**Published:** 2025-09-06

**Authors:** Lucas Sousa Salgado, Vinicius Domene, Thomas Peter Maahs, Rafael de Castro da Silva, Vanessa Carvalho de Oliveira, Renata Papatella Araujo, Carlos Takahiro Chone

**Affiliations:** Universidade Estadual de Campinas (UNICAMP), Departamento de Otorrinolaringologia, Campinas, SP, Brazil

## Introduction

Sinonasal Teratocarcinosarcoma (SNTCS) is a rare and aggressive tumor exhibiting a triphasic growth pattern: epithelial, mesenchymal, and neuroectodermal. It mainly affects the nasal cavity and paranasal sinuses, though nasopharyngeal and oral involvement may occur. Due to its rarity, fewer than 130 cases have been reported, making diagnosis and treatment challenging.^1^

The pathogenesis of SNTCS remains poorly understood, and its histopathological diagnosis can be complex, often requiring extensive tissue sampling and the use of immunohistochemical markers to differentiate it from other sinonasal tumors.^2^ Microscopically, SNTCS shows a complex mix of malignant epithelial, neuroectodermal, and mesenchymal elements. Immunohistochemistry assists in distinguishing these: cytokeratins and EMA mark epithelial areas; synaptophysin and S-100 identify neuroepithelial tissue; desmin or myogenin indicate mesenchymal differentiation.^3^^,^^4^ This histologic diversity may mimic other sinonasal tumors. Olfactory neuroblastoma shows neuroectodermal traits but lacks malignant epithelial or mesenchymal components. SNUC is a poorly differentiated carcinoma without triphasic features. Immature teratoma may resemble SNTCS but is less aggressive and affects younger patients.^4^ This case illustrates an uncommon presentation in a young adult and stresses the need for accurate pathology and multidisciplinary care.

## Case report

**Fig. 1:**
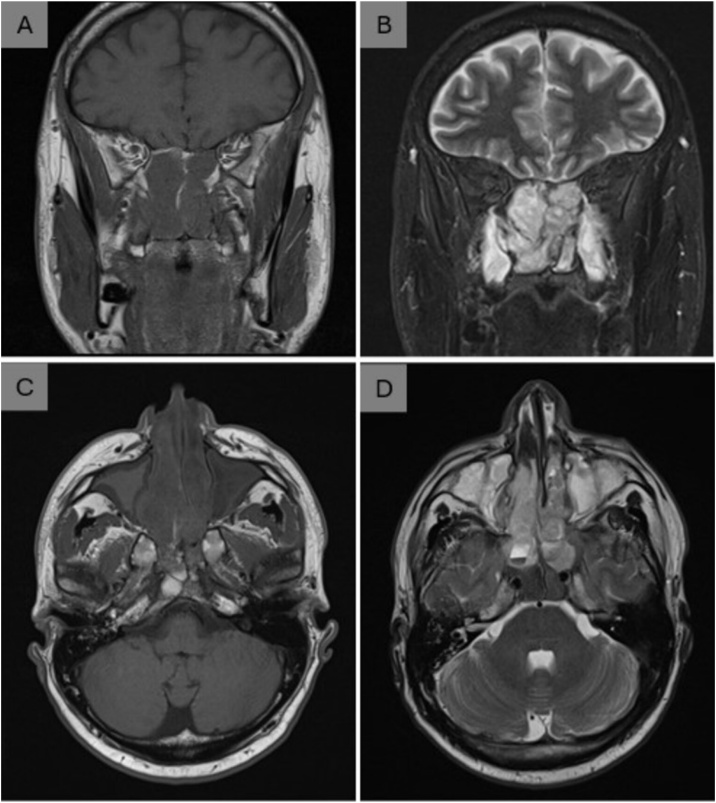
Coronal (A‒B) and axial (C‒D) MRI images showing a heterogeneous mass occupying the nasal cavities, with invasion of the ethmoid and sphenoid sinuses, skull base, and pushing the lamina papiracea.

A 49-year-old male presented with a five-month history of nasal obstruction, hyposmia, facial pain, and rhinorrhea. An outside otolaryngologist suspected a Killian’s polyp, and the patient underwent endonasal surgery with tissue sent for histopathology. Postoperatively, symptoms worsened, with persistent epistaxis and severe headaches. The initial biopsy was inconclusive. He was referred to our quaternary center, where CT and MRI revealed a large, heterogeneous mass filling the nasal cavities and invading adjacent structures. The lesion extended into the ethmoid without infiltration of lamina papiracea, medial wall of maxillary sinuses, and anterior wall of sphenoid sinuses bilaterally, with bone erosion involving the nasal septum, and skull base. Superiorly, it breached the cribriform plate with a tiny probable extension into the right frontal base. Posteriorly, it eroded the floor of the sella and encroached upon the sellar region. There was inferior extension into the posterior wall of nasopharynx and without invasion of both extraconal orbits, compressing the medial walls bilaterally (Fig. 1).

**Fig. 2:**
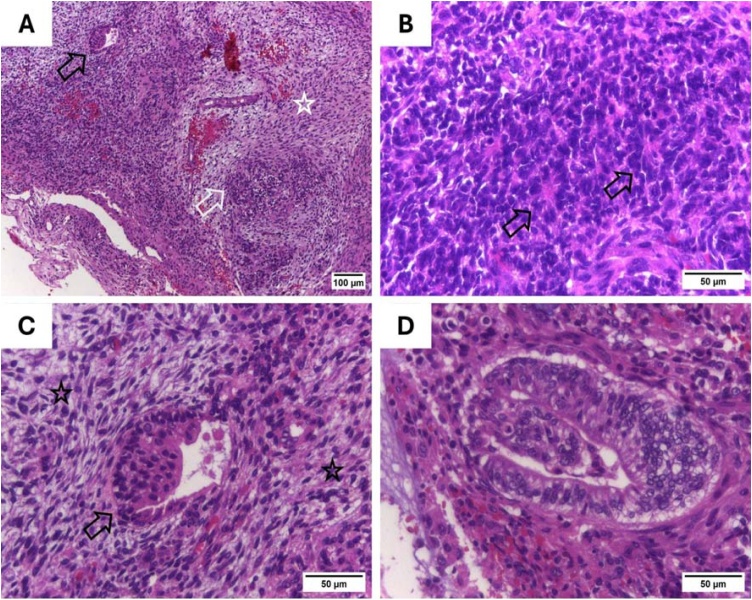
Histopathological analysis of a sinonasal teratocarcinosarcoma (Hematoxylin-Eosin). (A) Epithelial (black arrow), mesenchymal (star) and neuroepithelial elements (white arrow). (B) Primitive neuroepithelium with rosette formation (arrows). (C) Squamous epithelial element (arrow) surrounded by hypercellular fascicles of spindle cells from mesenchymal components (star). (D) Glandular element with a clear cell appearance.

**Fig. 3:**
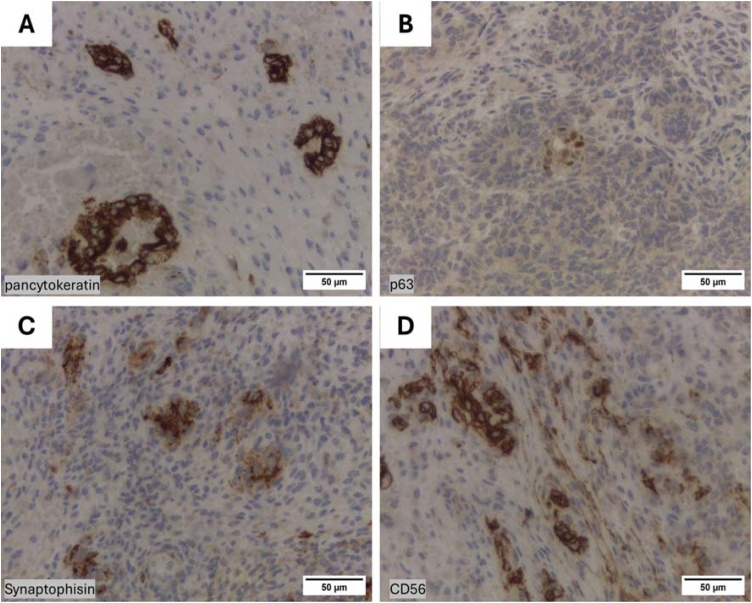
Representative immunohistochemistry of sinonasal teratocarcinosarcoma. Immunohistochemical analysis demonstrated pan-cytokeratin positivity in neuroepithelial areas and primitive glands (A). Rare epithelial cells showed p63 expression (B). Focal expression of Synaptophysin (C) and CD56 (D) was observed within the neuroepithelium.

A new biopsy was performed to clarify the lesion's nature. Pathology revealed a tumor with mesenchymal and neuroepithelial elements and sparse epithelial components (Fig. 2A). Mesenchymal areas showed hypercellular spindle cells in loose or dense fascicles, intermingled with immature neuroepithelial cells with hyperchromatic nuclei, occasionally forming rosettes (Fig. 2B). Epithelial nests of columnar or squamous cells (Fig. 2C), often forming clear cell glands (Fig. 2D). Immunohistochemistry showed pan-cytokeratin in neuroepithelial regions and primitive glands (Fig. 3A), and p63 in rare atypical epithelial cells (Fig. 3B). CK5/6 was negative. Synaptophysin (Fig. 3C), Chromogranin, CD56 (Fig. 3D), and S100 were focally positive in neuroepithelium. CD56 was also positive in sarcomatoid areas, and Desmin in rare spindle cells. Beta-catenin was negative throughout. SMARCA4 staining was unavailable. The findings confirmed a nasal teratocarcinosarcoma, a rare aggressive tumor with mesenchymal, neuroepithelial, and epithelial elements.

**Fig. 4:**
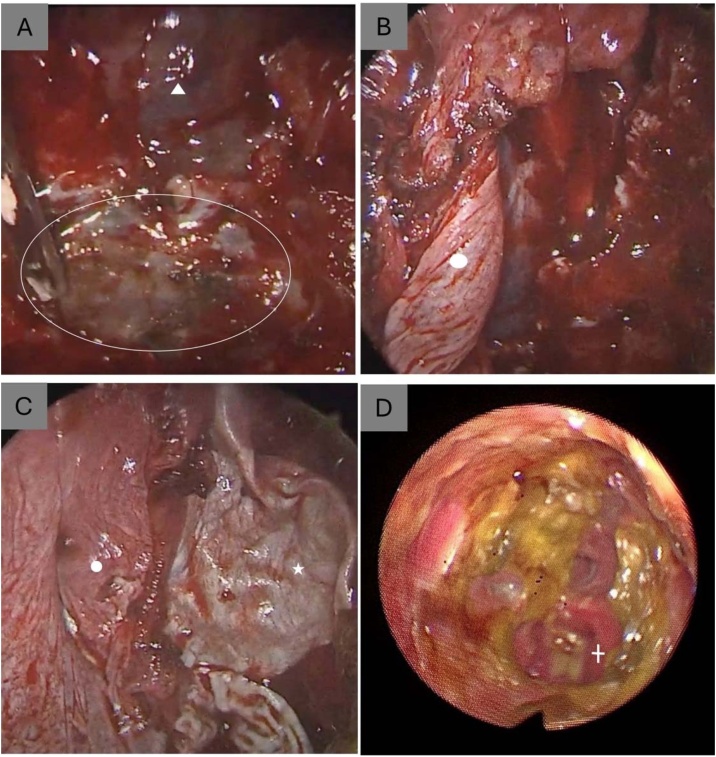
Intraoperative and postoperative images. (A) Exposure of the anterior skull base (white triangle) with dural resection and identification of the superior limit of the tumor (white oval outline). (B) Harvested pericranial and galeal flap (white circle) (C) Fascia lata graft harvested from the right thigh (white star). (D) Endoscopic view of the nasal cavity at 10-month postoperative follow-up, showing complete healing and no evidence of recurrence, indicating the location of the choana (white cross).

The tumor was completely resected via a purely endoscopic endonasal approach, involving nasal cavities nasopharynx, with bilateral extended medial maxillectomies with Denker approach and complete ethmoidectomies. Due to cribriform plate involvement, a transcribiform approach with dural resection and right frontal base dissection was performed. Skull base reconstruction used a pericranial and galeal flap harvested through a glabellar incision (combined endoscopic-open approach), through Draf III approach in frontal sinuses, supplemented by a fascia lata graft (Fig. 4). No external osteotomies or craniotomies were required. The surgical margins were negative within the resection field. Following surgery, the patient underwent adjuvant chemoradiotherapy to reduce the risk of recurrence. Chemotherapy with cisplatin 100 mg/m^2^ every 21 days, in three cycles, concurrently with radiotherapy. The third cycle was briefly delayed due to neutropenia but was completed. Radiotherapy was delivered using conventional external beam technique, with a total dose of 60 Gy.

In the early postoperative period, he developed acute bacterial meningitis, successfully treated with intravenous antibiotics. On follow-up, the patient remains asymptomatic and disease-free, with no signs of recurrence or metastasis on endoscopic and radiologic evaluation nor neurological sequelae. He continues under close multidisciplinary surveillance.

## Discussion

SNTCS poses diagnostic and therapeutic challenges due to its rarity and histologic complexity. It accounts for <1% of all cancers and ∼3% of head and neck malignancies, predominantly affecting males around 50-years-old, with symptoms like nasal obstruction, epistaxis, and occasionally visual loss or facial pain.^3^

In this case, diagnosis was established only after extensive histopathological analysis. Due to its aggressive behavior and reported recurrence rates up to 38%, a multimodal treatment approach was adopted. Our patient underwent complete endonasal endoscopic resection followed by concurrent chemoradiotherapy, aligning with evidence suggesting superior outcomes with trimodally therapy. A large systematic review of 127 cases found that 55% of patients treated with surgery and radiation, and 20% with surgery plus chemoradiation, had better survival than those treated with surgery alone (p < 0.001), although recurrence rates remained high across all groups.^2^

Our findings are consistent with those of Miller et al. (2020), who reported rapid progression and high recurrence in patients treated with surgery alone, while combined adjuvant chemoradiation (with cisplatin and IMRT to 66.6 Gy) showed more favorable disease control.^4^ Similarly, Yoon et al. reported a case of intracranial SNTCS recurrence during radiotherapy, which was subsequently managed with adjuvant chemotherapy using doxorubicin and ifosfamide, resulting in complete disease control at 9-month follow-up.^5^

In our case, despite cribriform plate and dural involvement, the tumor was completely resected with negative margins and no intraoperative complications. The patient subsequently underwent adjuvant chemoradiotherapy. At 10-months post-treatment, the patient remains recurrence-free, with no neurological deficits and preserved sinonasal function.

## Conclusion

This case underscores the rarity and complexity of SNTCS and the importance of a multidisciplinary approach for effective diagnosis and management. Surgical resection with reconstruction yielded a favorable outcome, highlighting the value of integrated care in specialized centers. Ongoing follow-up and further research on adjuvant therapies remain vital to improving prognosis.

## ORCID ID

Vinicius Domene: 0000-0002-8594-4961

Vanessa Carvalho de Oliveira: 0000-0002-3103-3433

## Declaration of competing interest

The authors declare no conflicts of interest.
